# Goldenhar Syndrome with Dextrocardia and Right Pulmonary Hypoplasia: An Unusual Association

**DOI:** 10.1155/2017/2625030

**Published:** 2017-03-09

**Authors:** Nagendra Chaudhary, Sandeep Shrestha, Hemant Kumar Halwai

**Affiliations:** ^1^Department of Pediatrics, Universal College of Medical Sciences, Bhairahawa 32900, Nepal; ^2^Department of Orthodontics, Universal College of Dental Sciences, Bhairahawa 32900, Nepal

## Abstract

Goldenhar syndrome (GS), a rare condition, occurring due to defect in development of first and second branchial arches, is characterized by a combination of various anomalies involving face, eyes, ears, vertebrae, heart, and lungs. The etiology of GS is not fully known, although various hypotheses have been proposed along with its genetic association and many other causes. Facial asymmetry and hypoplasia of the mandible are characteristic features of GS along with microtia and preauricular appendages and pits. Dextrocardia or pulmonary hypoplasia in GS has previously been reported separately. We report a 7-year-old female child of GS with combination of anomalies, dextrocardia, and pulmonary hypoplasia, which is a rare association.

## 1. Introduction

Goldenhar syndrome (GS) is a rare congenital malformation occurring due to defective development of first and second branchial arches which was first described in 1952 by a Swiss ophthalmologist Maurice Goldenhar. Later on, the terminology, oculoauriculovertebral (OAV) dysplasia was coined by Gorlin et al. in 1963 [1]. Hemifacial microsomia was included in the syndrome and termed as facioauriculovertebral syndrome (FAV) by Smith in 1988 [2]. The exact etiology of the syndrome is still unknown.

The disorder presents with complex manifestations including involvement of face, ears, eyes, and vertebrae with varying in severity [3].

Facial involvement includes unilateral facial hypoplasia, hypoplasia of maxillary, and mandibular and zygomatic bone. Eye involvement presents with epibulbar dermoid/lipodermoid, microphthalmia, anophthalmia, cataract, astigmatism, antimongoloid obliquity of palpebral fissures, blepharophimosis and colobomata of the upper eyelid, iris, and retina. Preauricular skin tags or blind fistulas, microtia, external ear malformations (dysplasia, asymmetries, aplasia, and atresia of the external meatus), and middle and internal ear anomalies are ear manifestations of the syndrome. Vertebral column anomalies (fused vertebrae, hemivertebrae, and scoliosis) and congenital heart disease are also associated with GS.

Pulmonary hypoplasia/aplasia with dextrocardia in GS has not been reported before from Nepal. This case of GS is probably the first case to the best of our knowledge showing the above-mentioned association.

## 2. Case Presentation

A 7-year-old girl, a product of nonconsanguineous parents, presented to pediatric outpatient department with complaints of deviation of face to left side and with progressively increasing mass noticed on left eye. Antenatal and birth history was uneventful. Developmental milestones were normal.

**Figure 1:**
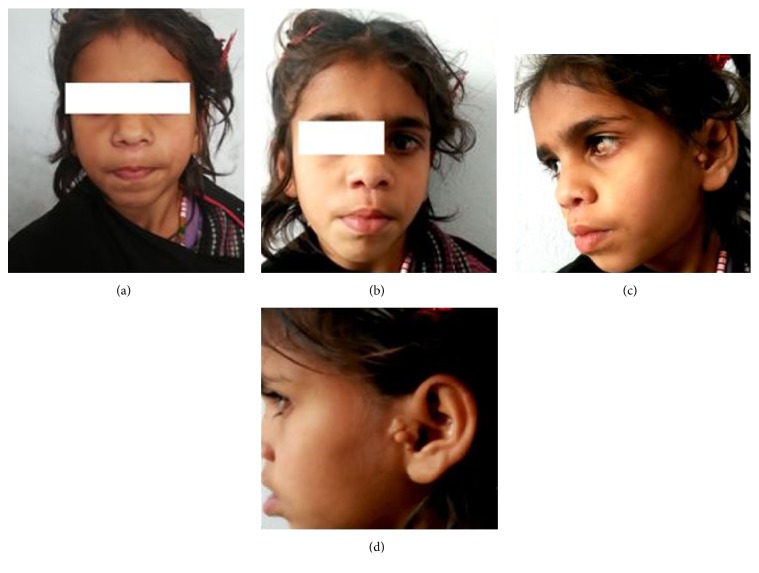
(a) and (b) showing facial deviation to left side with left mandibular hypoplasia; (c) showing left eye limbal dermoid; and (d) showing preauricular tag in left ear.

**Figure 2:**
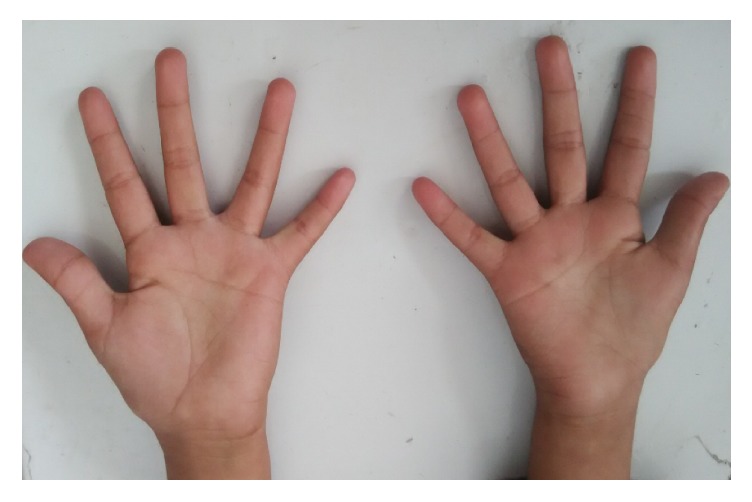
Showing atrophy of right thenar muscle.

Examination revealed deviation of face on left side suggesting hypoplasia of left mandible with high arched palate. Ear examination showed normal ears with left-sided preauricular tag. Hearing was normal. Eye examination showed left eye limbal dermoid (Figures 1(a)–1(d)). Limbs examination showed atrophy of left thenar muscles (Figure 2). Cardiovascular examination was suggestive of presystolic murmur. Respiratory examination showed decreased air entry on right side.

**Figure 3:**
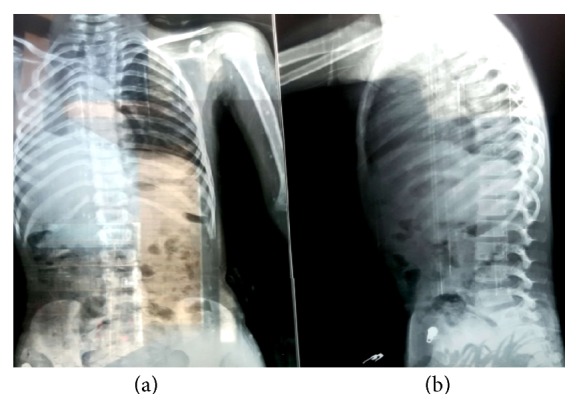
(a) X-ray showing dextrocardia, crowding of right-sided ribs, and tracheal shift to right side; (b) showing normal vertebrae.

Her height was 110 cm (<3rd centile) and weight was 13 kg (<3rd centile). Chest X-ray showed crowding of ribs on right hemithorax with dextrocardia. Skeletal survey did not show any vertebral deformities (Figure 3).

**Figure 4:**
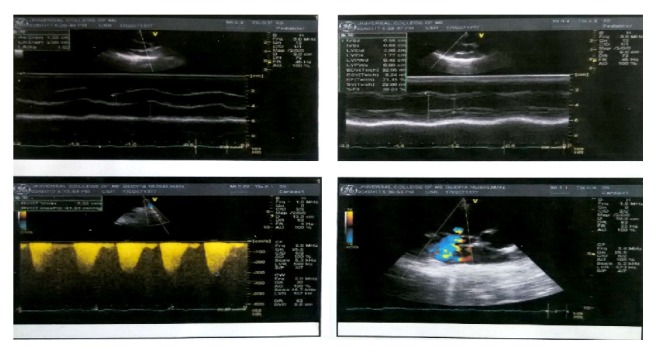
Showing echocardiographic findings.

Echocardiography was suggestive of situs solitus, dextrocardia, moderate-to-severe pulmonary stenosis (PSAP = 40 mm Hg), moderate-to-severe tricuspid regurgitation, dilated right atrium, and right ventricle with normal right and left ventricular systolic function (ejection fraction = 70%) (Figure 4). Karyotyping of the child could not be done due to unaffordability of the cost and its unavailability in our centre.

## 3. Discussion

Goldenhar syndrome is a rare congenital anomaly which consists of various malformations involving face, eyes, ears, and vertebrae [4].

GS has a prevalence rate of one per 3500–7000 live births with a male to female ratio of 3 : 2 [5, 6]. Although the exact etiology is unknown, various theories have been proposed for its occurrence. Reduced blood flow and focal haemorrhage in the developmental region of the first and second branchial arches in the blastogenesis period results in defect in mesoderm formation and its defective interaction with the neural crest cells [4].

Genetic defect, chromosomal anomalies, environmental effects, drug intake during pregnancy (cocaine, thalidomide, tamoxifen, and retinoic acid), maternal diabetes, and antepartum haemorrhage are possible causes that have been linked to the occurrence of GS [7–9]. Multiple chromosomal anomalies linked to GS are 3del(5p), del(6q), trisomy 7 mosaicism, del(8q) (161), trisomy 9 mosaicism (166), trisomy 18 (14,58), recombinant chromosome 18, del(18q), ring 21 chromosome, del(22q), dup(22q), trisomy 22, 49,XXXXX, 49,XXXXY, and 47,XXY. Familial cases in successive generation having history of consanguineous marriage have also been reported suggesting autosomal recessive, dominant, or multifactorial inheritance [6, 10–12]. We could not perform the karyotyping in the child due to its unavailability in our centre.

Facial abnormalities in GS include hypoplasia of mandible and maxilla, high arched palate, micrognathia, and dental malalignment. Facial asymmetry and hypoplasia of the mandible are characteristic features of GS. Our case had facial deviation to left side as shown in Figure 1 with mandible hypoplasia.

Ear abnormalities in GS vary from patients to patients. Microtia and preauricular appendages and pits, either alone or in combination, are necessary minimal criteria for diagnosing GS. Our patient had preauricular skin tags, although external ear and hearing were normal [13, 14].

Ocular anomalies are common in GS and include epibulbar dermoids, lipodermoids, microphthalmia, and upper palpebral colobomata. Limbal dermoids or lipodermoids are mainly seen in the infratemporal region of the eye [5, 15, 16]. Limbal dermoid was present in the right eye located in the temporal region in our case.

Vertebral anomalies reported in the literature include hypoplasia,and fusion or absence of certain vertebra, but there was not any vertebral defect in our patient [17]. We also noticed atrophy of the right thenar muscle as an associated anomaly in GS.

The reported frequency of cardiovascular defects ranges from 5% to 58% [15, 18, 19]. In a study conducted by Morrison et al. on 25 GS patients, 8 (32%) had cardiac defects which included ventricular septal defects in all of them [18]. The cardiac defects seen in our case were dextrocardia with valvular defects (pulmonary stenosis and mitral regurgitation) and dilatation of right atrium and ventricle.

Dextrocardia in GS has previously been reported by Shokeir (1977) and Wilson (1983) [20, 21].

Pulmonary agenesis/hypoplasia is considered a part of Goldenhar syndrome, and the syndrome with pulmonary agenesis is termed an expanded Goldenhar complex. Our case too had right pulmonary hypoplasia which was noticed in chest X-ray (shown in Figure 3(a)) as right-sided rib crowding with tracheal shift to same side. The association of pulmonary hypoplasia/agenesis with facial microsomia has been previously reported by few authors [22–26].

Ghimire et al. (2010) also reported left bronchopulmonary aplasia in an 11-year-old girl from Nepal [27]. Our case had both right lung hypoplasia and dextrocardia along with other features of GS. The importance of reporting this case is to focus on both anomalies in a child with GS.

## 4. Conclusion

The diagnosis of GS is mainly clinical which can be supported by radiological and laboratory tests. Most authors consider that the presence of microtia and preauricular tags along with facial asymmetry, mandible/maxilla hypoplasia, and epibulbar dermoid is necessary for diagnosis. Pulmonary (lung hypoplasia) and cardiovascular (dextrocardia and valvular defects) involvements although rare are associated with GS and have been reported sporadically. Proper examination and thorough investigations play an important role in identifying and management of associated anomalies in GS.
